# Multi-Component Protein Vaccine Induces a Strong and Long-Term Immune Response Against Monkeypox Virus

**DOI:** 10.3390/vaccines12121410

**Published:** 2024-12-13

**Authors:** Xiaolan Yang, Yakun Sun, Hongjing Gu, Deyu Li, Liangyan Zhang, Tao Li, Hui Wang

**Affiliations:** State Key Laboratory of Pathogens and Biosecurity, Academy of Military Medical Sciences, Beijing 100071, China

**Keywords:** monkeypox virus, protein vaccine, multicomponent, antigen, long-term, immune response

## Abstract

Background/Objectives: Since 2022, outbreaks of monkeypox have raised widespread concern and have been declared a public health emergency of international concern by the World Health Organization. There is an urgent need to develop a safe and effective vaccine against the monkeypox virus (MPXV). Recombinant protein vaccines play a significant role in the prevention of infectious diseases due to their high safety and efficacy. Methods: We used the A29, E8, M1, A35, and B6 proteins of MPXV as candidate antigens to generate a panel of multi-component MPXV vaccine candidates, which were administered subcutaneously to immunize mice. Results: The results showed that the vaccine candidates Mix-AEM, Mix-AEMA, Mix-AEMB, and Mix-AEMAB effectively elicited strong neutralizing antibody responses and demonstrated significant protection against vaccinia virus (VACV) infection in a murine model. The vaccine candidate Mix-AEM induced significantly higher levels of neutralizing antibodies, cellular immunity capacity, and virus clearance compared to the vaccine candidate Mix-AE (lacking M1). Single-component immunization showed that M1 induced higher levels of neutralizing antibodies than A29 and E8. These results indicated that M1 is a critical and essential antigen in the MPXV vaccine. The number of cells secreting IFN-γ was significantly increased in the Mix-AEMA and Mix-AEMAB groups compared to the A35-deficient vaccine candidates, demonstrating the important role of A35 in inducing IFN-γ secreting. In addition, the neutralizing antibodies induced by these multi-component vaccine candidates were maintained at high levels six months after the third immunization. Conclusions: In summary, this study lays the groundwork for combining antigens to develop multi-component subunit vaccines.

## 1. Introduction

The monkeypox virus (MPXV), which can cause zoonotic disease, was first recognized and isolated from monkeys in 1958. The first known human cases were reported in the Democratic Republic of the Congo in 1970 [1]. Since 2022, cases of MPXV infection have been reported in monkeypox non-endemic countries and have rapidly spread to six WHO-covered regions, including Europe and the Americas and so on, ultimately leading to its declaration by the World Health Organization (WHO) as a “public health emergency of international concern”, and the alert was lifted in May 2023. However, last year, there was a significant increase in reported instances, and the total number of cases reported this year has already exceeded last year’s figure, with over 14,000 cases and 524 deaths [2]. Under these conditions, in August 2024, the monkeypox outbreak was reclassified as a “Public Health Emergency of International Concern”.

Common symptoms of MPXV infection include fever, headache and myalgia, swollen lymph nodes [3,4], and rashes that appear following the onset of fever [5]. In severe cases, MPXV infection can affect the respiratory and gastrointestinal tracts, leading to restrictions on breathing, drinking and eating [6], and even encephalitis [7], septicemia [8,9], and severe eye infections [7]. Of note, some atypical clinical symptoms were observed in this epidemic, characterized by lesions confined to the genital and perianal [10], so this phenomenon that may be associated with sexual transmission. To date, the outbreak of MPXV infection across multiple countries has predominantly occurred within sexual networks of men who have sexual relations with other men (MSM) [2,3,11].

MPXV is classified within the genus Orthopoxvirus of the Poxviridae family, characterized as an enveloped virus possessing a double-stranded DNA genome approximately 197 kilobases in length, which encodes about 190 distinct proteins. MPXV is divided into two distinct clades: clade I, which includes subclades Ia and Ib, and clade II, which encompasses subclades IIa and IIb. In 2022–2023, a global outbreak of mpox was caused by the clade IIb strain; however, the currently predominant epidemic strain is identified as ‘branch Ib’, which is more susceptible and lethal than strain IIa. The proteins encoded by most members of the genus Orthopoxvirus, including vaccinia virus (VACV), variola virus (VARV), cowpox virus (CPXV), and rabbitpox virus (RPXV), are highly conserved [12,13]. A previous study involving 215 cases of monkeypox indicated that individuals who had received the smallpox vaccine demonstrated an 85% efficacy in protecting against MPXV infection [14]. The U.S. Food and Drug Administration (FDA) has approved JYNNEOS and ACAM2000 [15,16] for preventing MPXV infection. However, ACAM2000 may be associated with adverse effects, including dermatitis and even myocarditis and pericarditis after vaccination [17]. In contrast, JYNNEOS is a third-generation, attenuated, non-replicating vaccine derived from modified vaccinia Ankara (MVA), which is characterized by a better safety profile; however, further study is required to assess its efficacy against MPXV. In addition, recent clinical data have shown that JYNNEOS and ACAM2000 provide limited protection against MPXV due to the low level of cross-reactive neutralizing antibody (NAb) [18,19]. A safe and efficient MPXV vaccination is urgently required.

Recombinant protein vaccines, with the advantages of high safety and greater immunogenicity, offer an effective technological solution for the management and prevention of infectious diseases. Cells infected with the monkeypox virus (MPXV) exhibit the presence of both intracellular mature virus (IMV) and extracellular enveloped virus (EEV) [20]. Previous studies of orthopoxvirus have confirmed that L1R, D8L, and A27L are IMV-neutralizing antibody targets; B5 and A33 are known EEV-neutralizing antibody targets [21,22,23,24,25]; and the above proteins correspond to the M1, E8, A29, A35, and B6 of MPXV, respectively. Recent studies have shown that multivalent subunit vaccines targeting M1, E8, A29, A35, and B6 of MPXV induce a high level of immune response [12,26,27,28,29]. The proteins present on the surfaces of IMV and EEV exhibit significant compartmentalization, and research indicates that the immune response induced by a single antigen is not as strong as that induced by combinations of multiple antigens [30,31]. In this study, we used M1, E8, A29, A35, and B6 as target antigens, which were combined in different ways, to immunize mice and to find an optimal combination of antigens by detecting the antibody level and the protective effect of the attack, so as to provide scientific clues for the rational development of MPXV vaccines.

## 2. Materials and Methods

### 2.1. Cells and Viruses

Vero cells were cultured at 37 °C with 5% CO_2_ in Dulbecco’s modified Eagle’s medium (DMEM, Gibco, New York, NY, USA), supplemented with 1% penicillin–streptomycin (PS, Gibco, New York, NY, USA) and 10% heat-inactivated fetal bovine serum (FBS, Gibco, New York, NY, USA). These Vero cells were then utilized to produce the VACV Tian Tan strain. The cells were infected with VACV at a multiplicity of infection (MOI) of 0.1 and incubated for 72 h at 37 °C. Following this period, the cells underwent three cycles of freeze–thawing before being centrifuged at 4000× *g* for 5 min to eliminate cell debris. The resulting supernatant was collected and stored at −80 °C; after which, viral titers were determined using a standard plaque assay.

### 2.2. Protein Expression, Purification, and Identification

The protein sequences for MPXV A29, E8, M1, A35, and B6 were obtained from the gene bank, using the sequence of MPXV_USA_2022_MA001 (GenBank accession number ON563414.2). The coding sequences for MPXV A29, E8, M1, A35, and B6, each containing a His-tag at the C-terminus, were synthesized by Sangon Biotech in Shanghai, China. A29 and E8 were cloned into either the pET-9a or pET-26b plasmids and subsequently transformed into *E. coli* BL21 (DE3) competent cells. Meanwhile, M1, A35, and B6 were cloned into the pCDNA3 plasmid and transformed into HEK 293 cells. The five outer membrane proteins of MPXV were then expressed and purified using Ni-NTA affinity chromatography with an AKTA purification system (Cytiva, Marlborough, MA, USA). Following purification, the proteins were dissolved in PBS buffer, and their concentrations were measured using the BCA method. For Western blot analysis, the purified proteins were separated using 12% SDS-PAGE and transferred to a 0.4 µm polyvinylidene fluoride (PVDF) membrane (Immobilon, MERCK, Rahway, NJ, USA). The membrane was blocked with a 5% skim milk solution at room temperature for two hours; after three washes, specific antibodies against A29 (Vazyme, Nanjing, China), E8 (AntibodySystem, Strasbourg, France), M1 (AntibodySystem, Strasbourg, France), A35 (Sinorganic Biological, Beijing, China), and B6 (AntibodySystem, Strasbourg, France) were incubated overnight at 4 °C. Subsequently, the membrane was incubated with a HRP-conjugated goat anti-mouse/rabbit antibody (Transgen Biotech, Beijing, China) at 37 °C for 60 min, and the results were visualized using chemiluminescent HRP substrates.

### 2.3. Mouse Immunization and Challenge Protocol

**Figure 1:**
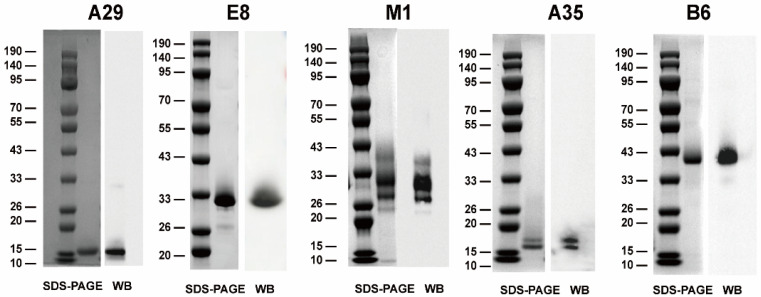
Expression and characterization of recombinant MPXV proteins A29, E8, M1, A35, and B6 using SDS-PAGE (**left**) and Western blot (**right**).

All animal procedures were approved by the Animal Experiment Committee of Laboratory Animal Center, Academy of Military Medical Sciences (AMMS) (approval number: IACUC-IME-2023-037). Female BALB/c mice aged between 4 and 6 weeks were purchased from SPF (Beijing, China) Biotechnology Co., Ltd. and were immunized subcutaneously with Mix-AE (A29 + E8), Mix-AEM (A29 + E8 + M1), Mix-AEMA (A29 + E8 + M1 + A35), Mix-AEMB (A29 + E8 + M1 + B6), and Mix-AEMAB (A29 + E8 + M1 + A35 + B6), containing 20 μg of total recombinant proteins, as shown in Figure 1, respectively. Boosting was performed on days 14 and 28. The placebo group of mice received subcutaneous injections of the SP01 adjuvant, which contained squalene, polyether, and castor oil, mixed in a 1:1 *v/v* with the PBS, as previously described [32]. Serum samples were obtained at 14, 28, and 42 days following the initial immunization. Five mice from each group were subjected to an intranasal challenge with 4 × 10^5^ PFU of VACV (strain Tian Tan) 71 days after the initial immunization. The mice were subsequently euthanized three days post-infection, and lung tissues were harvested for the evaluation of the viral load and histopathological analysis.

### 2.4. Enzyme-Linked Immunosorbent Assay (ELISA)

The IgG antibody titers against MPXV-specific antigens were determined by ELISA. Briefly, ELISA plates were coated overnight at 4 °C with 1 µg/mL of VACV. After three washes with PBS containing 0.05% Tween 20, the plates were blocked for one hour using TBST (Tris-buffered saline with Tween 20) supplemented with 3% FBS. Heat-inactivated serum, diluted in three-fold serial dilutions, was then added and incubated for one hour at 37 °C. Following another wash, a horseradish peroxidase (HRP)-conjugated goat anti-mouse IgG antibody (diluted 1:10,000; TransGen Biotech, Beijing, China) was added to the wells and incubated for an additional hour at 37 °C. The plates were washed four times before being treated with the substrate tetramethyl-benzidine (TMB; Solarbio, Beijing, China). The color reaction was stopped with 2 N sulfuric acid, and the absorbance was measured at 450/630 nm using a microplate reader (Thermo Mutiskan, Thermo Fisher, Waltham, MA, USA). Endpoint titers were determined according to the manufacturer’s guidelines.

### 2.5. Plaque Reduction Neutralization Test

The 50% plaque reduction neutralization test (PRNT_50_) was utilized to assess the titers of neutralizing antibodies. The methods were conducted as in previous studies [26,28]. Vero E6 cells were maintained in a 24-well culture plate. The next day, serial two-fold dilutions of serum were incubated with 100 plaque-forming units (PFUs) of VACV at a temperature of 37 °C for 2 h. Following this incubation, the serum–virus mixture was transferred to duplicate wells and incubated for an additional 2 h. Following the incubation, the supernatant was removed, and the cell monolayers were covered with low-melting agar, creating a medium that consisted of DMEM supplemented with 1% low-melting agarose, 2% fetal bovine serum (FBS), and 50 U/mL penicillin–streptomycin. After 72 h, the plates were fixed with 4% paraformaldehyde for 30 min, and the cell monolayers were stained with 1% crystal violet. The resulting plaques were enumerated, and the PRNT_50_ titers were determined using the Spearman-Karber method.

### 2.6. IFN-γ ELISpot Assay

On day 56 following the initial immunization, five mice from each group were euthanized. Spleens were collected, and splenic lymphocytes were extracted using mouse lymphocyte isolation solution (DAKEWE, Shenzhen, China). The Mouse IFN-γ ELISpot kit (DAKEWE, Shenzhen, China) was utilized to conduct the ELISpot assay, following the provided protocol. Briefly, 2 × 10^5^ cells were added to each well of a 96-well plate and stimulated for 20 h with a peptide mixture consisting of A29, E8, M1, A35, and B6 at a total concentration of 10 µg/mL. Phorbol-12-myristate-13-acetate (PMA) and R10 served as positive and negative controls, respectively. Following the incubation period, deionized water was added to the cells, and they were maintained at 4 °C for 10 min to induce cell lysis. After performing six washes with a 1× washing buffer, 100 µL of a 1× biotinylated antibody was added to the cells and allowed to incubate at 37 °C for one hour. This was followed by the addition of 100 µL of a 1× streptavidin antibody, which was similarly incubated at 37 °C for one hour. Subsequently, AEC working solution was applied to the wells and incubated at 37 °C for 5 to 30 min. Finally, the AEC solution was removed, and the wells were rinsed with deionized water to stop the reaction. The spots were analyzed and counted using an ImmunoSpot CTL reader (Cellular Technology Limited, Cleveland, OH, USA) and represented as the number of cells producing IFN-γ per 2 × 10^5^ splenocytes. Each group consisted of 4 samples, with 2 replicates for every sample.

### 2.7. Quantitative Real-Time PCR and Measurement of Infectious Virus Particles

The lung tissue samples were measured, homogenized, and clarified through centrifugation at 8000 rpm for 10 min. A standard plaque assay, described previously [26], was employed to identify infectious viral particles present in the supernatants using Vero E6 cells. Viral DNA was extracted using Viral RNA/DNA Extraction Kits (TIANGEN, Beijing, China). Additionally, quantitative PCR (qPCR) was conducted with qPCR Mix (Takara, Beijing, China) using the following primers and probes: VACV-F (5′-GGCAATGGATTCAGGGATATAC-3′) and VACV-R (5′-ATTTATGAA-TAATCCGCCAGTTAC). The PCR process was performed on a LightCycler^®^ 96 Instrument (Roche Diagnostics Ltd., Basel, Switzerland).

## 3. Results

### 3.1. Expression and Purification of Recombinant MPXV Proteins

In order to create a recombinant MPXV vaccine candidate, five distinct MPXV outer membrane proteins were expressed and purified individually. These included the IMV-derived antigens A29, E8, and M1 and the EEV-derived antigens A35 and B6 (Figure 1), based on the sequence MPXV_USA_2022_MA001 (GenBank accession number ON563414.2). Subsequently, the purified recombinant proteins A29, E8, M1, A35, and B6 were characterized by SDS-PAGE and Western blot, which revealed that the bands of these proteins approximated the predicted protein molecular weights (Figure 1).

### 3.2. Multiple Multi-Component Protein Vaccines Elicited Robust Antibody Responses at High Titers

**Figure 2:**
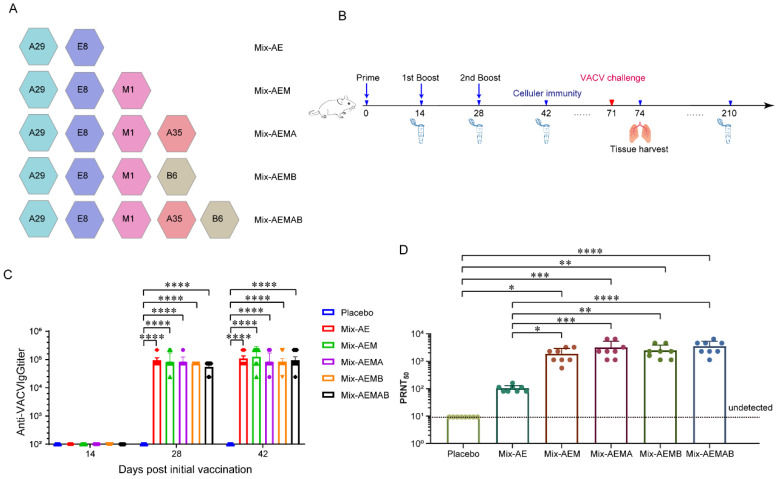
Multi-component protein vaccine elicited a robust immune response in BALB/c mice. The mice were administered either the protein vaccine or a placebo, followed by a booster immunization with the same dosage two weeks later. Serum samples were collected at the specified time after immunization. (**A**) Experimental groups. (**B**) Immunization and challenge schematic diagram. (**C**) Antibody titers specific to VACV were assessed using ELISA. (**D**) The levels of neutralizing antibodies against VACV were measured through the PRNT assay. The results are presented as the mean ± SD. Statistical analysis was performed using Student’s *t*-test for normally distributed data or one-way ANOVA followed by Dunnett’s multiple comparison test (* *p* < 0.05, ** *p* < 0.01, *** *p* < 0.001, and **** *p* < 0.0001).

To determine the optimal combination of different recombinant protein vaccine formulations, five candidate formulations were prepared, each containing an equal amount of recombinant protein antigen and designated Mix-AE (A29 + E8), Mix-AEM (A29 + E8 + M1), Mix-AEMA (A29 + E8 + M1 + A35), Mix-AEMB (A29 + E8 + M1 + B6), and Mix-AEMAB (A29 + E8 + M1 + A35 + B6) (Figure 2A). Groups of mice aged between four and six weeks were immunized subcutaneously three times, with a 14-day interval between each administration (Figure 2B). The SP01 (a MF-59-like squalene adjuvant) adjuvant was employed, while the placebo groups were administered SP01 alone. Serum samples were obtained 14 days following each immunization. Serum IgG titers were determined by indirect ELISA using vaccinia virus (VACV)-coated plates. After the prime immunization, binding antibodies were either undetectable or minimal. As shown in Figure 2C, following the second immunization, there was a marked elevation in IgG titers across all vaccination groups. Furthermore, the IgG titers measured following the second vaccination in each group were similar to those after the third immunization, while no antibodies were detected in the placebo group of mice. These results suggest that two immunizations with AE could provide a sufficiently high IgG antibody response.

Then, the concentration of neutralizing antibodies was evaluated using the PRNT test with VACV in serum samples collected 42 days following the initial immunization (Figure 2D). The results showed that neutralizing antibody titers of the four groups, including Mix-AEM (A29 + E8 + M1), Mix-AEMA (A29 + E8 + M1 + A35), Mix-AEMB (A29 + E8 + M1 + B6), and Mix-AEMAB (A29 + E8 + M1 + A35 + B6), were significantly higher than those of the Mix-AE (A29 + E8) and placebo groups, with median values of 1:1859 (Mix-AEM), 1:3130 (Mix-AEMA), 1:2460 (Mix-AEMB), and 1:3483 (Mix-AEMAB). The neutralizing antibody observed in in the Mix-AE group (1:102) was slightly higher than that in the placebo group (undetected).

### 3.3. M1 Elicited Greater Levels of Neutralizing Antibodies in Contrast to the A29 and E8

**Figure 3:**
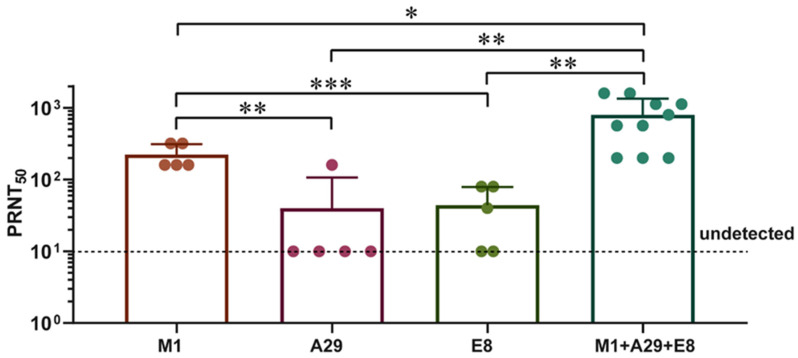
M1 induced higher levels of neutralizing antibody potency than A29 and E8. BALB/c mice received immunization using 10 µg of vaccines composed of a single protein component: M1; A29; E8; or a trivalent vaccine consisting of M1, E8, and A29. Alum was used as an adjuvant, and the same dose was administered for booster immunization two weeks later. Serum samples were obtained 14 days following the last immunization, and the levels of neutralizing antibodies against VACV were assessed using the PRNT assay. Data are presented as the mean ± SD. The analysis of the data was performed using Student’s *t*-test for normally distributed data or one-way ANOVA, followed by Dunnett’s multiple comparison test (* *p* < 0.05, ** *p* < 0.01, and *** *p* < 0.001).

To determine the role of three antigens in Mix-AEM, BALB/c mice were immunized with 10 µg of single-component protein vaccines (M1, A29, or E8), and immunization with Mix-AEM was used as a control. The results (Figure 3) showed that the neutralizing antibody titers of the M1 group were significantly higher than those of the A29 or E8 groups (*p* < 0.01), although slightly lower than those of the Mix-AEM group (*p* < 0.05), with median values of 1:240 (M1), 1:46 (A29), 1:50 (E8), and 1:865 (Mix-AEM), respectively. These results demonstrated that M1 plays an important role in inducing humoral immunity.

### 3.4. Multiple Multi-Component Protein Vaccines Induced Cellular Immunity in Mice

**Figure 4:**
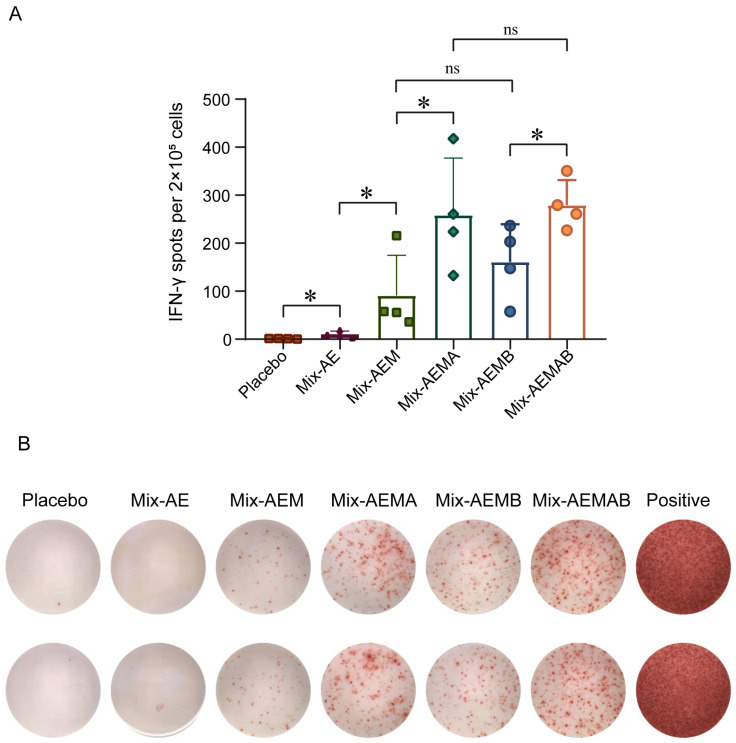
Cellular immune responses of multi-component protein vaccine immunization in mice. (**A**,**B**) The number of T cells producing IFN-γ was evaluated using ELISPOT assays. Four mice from each group were euthanized, and their splenocytes were collected 2 weeks after the second boost. Single-cell suspensions were then stimulated with peptide pools composed with A29, E8, M1, A35, and B6 peptide pools for 20 h. *n* = 4/group. Each sample was tested twice. Data are presented as the mean ± SD. The analysis of the data was performed using Student’s *t*-test for normally distributed data or one-way ANOVA, followed by Dunnett’s multiple comparison test (ns, not significant; * *p* < 0.05).

T cell responses were evaluated using the IFN-γ ELISpot assay. Spleens from mice were harvested two weeks following the third immunization. Splenocytes were isolated and subsequently stimulated with a composite peptide pool consisting of A29, E8, M1, A35, and B6 to assess the cellular immune responses (Figure 4A,B). The results demonstrate that the vaccine groups Mix-AE, Mix-AEM, Mix-AEMA, Mix-AEMB, and Mix-AEMAB induced IFN-γ secretion with an average of 10, 91, 258, 161, and 279 spots per 10^5^ cells, respectively. In contrast, IFN-γ spots were barely detectable in the placebo group. Notably, the numbers of IFN-γ spots were significantly increased in the Mix-AEM, Mix-AEMA, Mix-AEMB, and Mix-AEMAB groups compared with the Mix-AE group (*p* < 0.01). The numbers of IFN-γ spots were significantly higher in the Mixed-AEMA and Mixed-AEMAB groups compared to the Mixed-AEM group (*p* < 0.01). Conversely, no significant difference was observed between the Mixed-AEM and Mixed-AEMB groups (*p* > 0.05). These results suggest that A35 is able to induce cellular immunity more efficiently, whereas B6 has no role in inducing cellular immunity.

### 3.5. Multiple Multi-Component Protein Vaccines Inhibited VACV Proliferation

**Figure 5:**
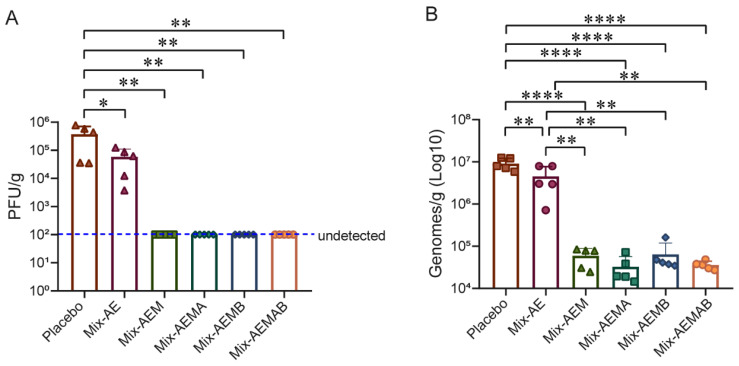
The protection against the challenges of VACV in multi-component protein vaccine immunization in mice. (**A**) The titers of VACV in the lungs were assessed through a standard plaque assay in Vero E6 cells. (**B**) Viral genome copies in lung were determined by qPCR. Five mice in every group were challenged with VACV, *n* = 5/group. Data are presented as mean ± SD. The analysis of the data was performed using Student’s *t*-test for normally distributed data or one-way ANOVA, followed by Dunnett’s multiple comparison test (ns, not significant; * *p* < 0.05; ** *p* < 0.01; **** *p* < 0.0001).

The VACV Tian Tan strain, which was highly homologous to MPXV_USA_2022_MA001, was employed to assess the protective efficacy of the multiple recombinant MPXV vaccines in a murine model. On day 71 post-initial immunization, all mice received an intranasal administration of 4 × 10⁵ PFU of VACV (Figure 2A), then were euthanized on day 3 of virus infection, and VACV replication in the lungs was quantified using standard plaque assays (see Figure 5A) and qPCR (Figure 5B). We found that high concentrations of infectious virus particles and viral DNA were observed in the placebo group, with peak levels of infectious virus particles and viral DNA reaching 7.78 × 10^5^ PFU/g and 1.25 × 10^7^ copies/g, respectively. As expected, both DNA genome levels and viral particles in the vaccine groups were lower than in the placebo group. Viral DNA in the Mix-AEM, Mix-AEMA, Mix-AEMB, and Mix-AEMAB groups were found to be significantly reduced in comparison to those in the Mix-AE group, while no infectious virus particles were detected in these four vaccine groups. The results indicate that all candidate vaccines were effective in reducing VACV replication in the lungs. However, the Mix-AE vaccine showed limited ability to inhibit viral replication due to lower levels of neutralizing antibodies and cellular immunity. In contrast, the other four vaccine groups demonstrated a significant protective effect against the VACV challenge in the murine model.

### 3.6. Multiple Multi-Component Vaccines Induce a Long-Term Immune Response

**Figure 6:**
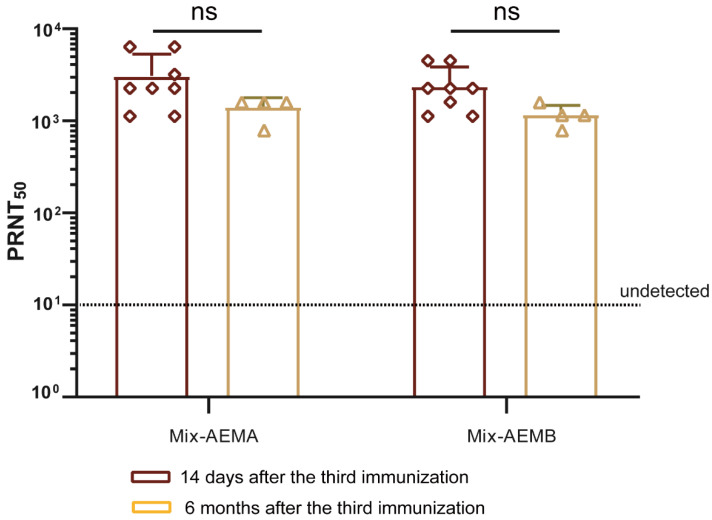
Multi-component vaccine candidates induce long-term immune response. BALB/c mice were immunized with the vaccine candidates Mix-AEMA or Mix-AEMB, and the doses and schedules were the same as shown in Figure 2B. Serum samples were obtained at 14 days (*n* = 8/group) and 6 months (*n* = 4/group) following the third immunization, and the levels of neutralizing antibodies against VACV were assessed using the PRNT assay. Data are presented as mean ± SD. The analysis of data was performed using Student’s *t*-test for normally distributed data or one-way ANOVA, followed by Dunnett’s multiple comparison test (ns, not significant).

To determine the duration of the vaccine immunity response, serum samples from mice immunized with vaccine candidates Mix-AEMA and Mix-AEMB were collected six months following the third immunization, and neutralizing antibody titers were tested. The findings indicated that the titers of the neutralizing antibody in the Mix-AEMA and Mix-AEMB groups were sustained at a high level six months post the third immunization, with median values of 1:1400 (Mix-AEMA) and 1:1165 (Mix-AEMB), respectively (Figure 6). Furthermore, these titers were not significantly lower in comparison to the levels observed 14 days following the third immunization. These results demonstrated that these multi-component vaccine candidates could induce a long-term immune response.

## 4. Discussion

With the global spread of the MPXV, it is evident that more safe and effective vaccines are needed to prevent the MPXV virus infection. In light of the experience in the development of COVID-19 vaccines, attention was quickly drawn to the development strategy of a MPXV vaccine founded upon mRNA and protein subunit vaccine technology, which has been demonstrated to be more efficacious against COVID-19 [33,34,35,36].

Here, a set of multiple recombinant protein vaccine candidates targeting MPXV was explored, comprising distinct mixtures of IMV surface proteins (M1, E8, and A29) and EEV surface proteins (A35 and B6). M1, E8, A29, A35, and B6 have been extensively studied as effective targets for subunit vaccines that protect mice against the Orthopoxvirus challenge. A mRNA vaccine study published in 2023 showed that mRNA the vaccine candidates encoding six (M1, H3L, A29, E8, B6, and A35) mpox virus antigens provided effective cross-protection against the lethal VACV challenge in a mouse model [29]. Another study published in the same year also devised a set of multi-component mRNA vaccines against MPXV using M1, E8, A29, A35, and B6 as the antigens. The findings indicated that the AR-MPXV5 vaccine (five components) and the AR-MPXV4a vaccine (four components) effectively elicited a robust neutralizing antibody response and demonstrated significant protective efficacy against VACV infection in a murine model [26].

The findings of this study demonstrated that the trivalent vaccine candidate Mix-AEM, the quadrivalent vaccine candidates Mix-AEMA and Mix-AEMB, and the pentavalent vaccine candidate Mix-AEMAB successfully induced elevated levels of neutralizing antibodies, as well as provided robust protection against challenges with VACV. Especially, the vaccine candidate Mix-AEM induced a higher level of neutralizing antibodies and cellular immunity capacity compared with the vaccine candidate Mix-AE, a bivalent vaccine lacking M1 (Figure 2 and Figure 4). On the other hand, following the VACV challenge, significantly reduced levels of viral DNA replication were noted in the Mix-AEM group, while elevated levels were observed in the Mix-AE group, approximately 75-fold higher than those in the Mix-AEM group (Figure 5). Similar results were also shown in previous mRNA vaccine studies, and the absence of M1 in the vaccine led to a notable decline in the production of neutralizing antibodies, along with a greater degree of weight loss following a challenge with VACV [26]. The findings indicate that M1 represents a crucial primary target of the MPXV vaccine.

A recent study has indicated that vaccination with M1 mRNA provides 100% protection against the VACV challenge [13]. However, the M1 recombinant protein has been found to offer only limited protection against the poxvirus challenge [37]. These findings suggest that there may be differences in the design of mRNA vaccines versus recombinant protein vaccines. In our study, we observed that M1 induced significantly higher levels of neutralizing antibodies than A29 and E8 when immunized with a single component (Figure 3). Combined immunization with E8, A29, and M1 (10 µg total protein) resulted in higher neutralizing antibody potency than immunization with M1 (10 µg), A29 (10 µg), or E8 (10 µg) alone. These results suggest that M1 is a critical and essential antigen in the MPXV vaccine and that E8 and A29 have a potentiating effect on M1. The mechanism of the synergistic enhancement of immunity between these proteins requires further investigation.

IFN-γ is a crucial factor in the host response to poxvirus infections. Mature Th1 cells secrete IFN-γ, which has direct antiviral effects and enhances the Th1 response [28]. Previous studies [9] indicate that spleen IFN-γ production following ectromelia virus (ECTV) infection is associated with susceptibility to ECTV. Low IFN-γ producers exhibit increased susceptibility, whereas high producers demonstrate resistance to viral infection. In this study, the quantity of mouse splenocytes that were able to secrete IFN-γ was quantified using an ELISpot assay. The results demonstrated that the findings revealed that the number of IFN-γ-secreting cells was markedly more in the A35-added Mix-AEMA and Mix-AEMAB groups compared to the A35-deficient vaccine candidates. This finding is consistent with previous reports by Asaf Poran et al. [13], which showed that A35 drives the most robust T cell response compared to M1 and B6. The mechanism by which A35 promotes IFN-γ secreting is still being explored.

A further significant finding was that the neutralizing antibody potency remained at a high level in both the Mix-AEMA and Mix-AEMB groups when tested six months after the third immunization (Figure 6), indicating that the vaccine candidates could induce a long-term immune response. However, due to the limited number of animals, the long-term observations of the other groups were not carried out.

## 5. Conclusions

In conclusion, we have identified multiple MPXV antigen combinations that elicit strong neutralizing antibody responses, as well as confer significant protection against infection in murine models. Our findings demonstrate the indispensable role of M1 and A35 in the induction of humoral and cellular immunity, respectively. We have also proposed that A29 and E8 have auxiliary synergistic effects on the M1-induced generation of neutralizing antibodies. The findings of this study offer a significant foundation for the design and development of recombinant protein vaccines targeting MPXV.

## Data Availability

All data presented are available in the manuscript.
